# Discovery of a Novel Anticoagulant Cystine Knot Peptide from Spider Venom Gland Transcriptome

**DOI:** 10.3390/ijms262010154

**Published:** 2025-10-19

**Authors:** Jinai Gao, Di Yang, Wanting Wang, Xiaoshan Huang, Ruiyin Guo, Kaixun Cao, Qiumin Lu, Ziyi Wang, Ren Lai, Juan Li

**Affiliations:** 1 Engineering Laboratory of Peptides of Chinese Academy of Sciences, Key Laboratory of Bioactive Peptides of Yunnan Province, KIZ-CUHK Joint Laboratory of Bioresources and Molecular Research in Common Diseases, National Resource Center for Non-Human Primates, National Research Facility for Phenotypic & Genetic Analysis of Model Animals (Primate Facility), State Key Laboratory of Genetic Evolution & Animal Models, Sino-African Joint Research Center, and New Cornerstone Science Laboratory, Kunming Institute of Zoology, Chinese Academy of Sciences, No. 17 Longxin Road, Kunming 650201, China; 2 School of Molecular Medicine, Hangzhou Institute for Advanced Study, Hangzhou 310024, China; 3 Institute of Chemistry, Chinese Academy of Sciences, Beijing 100190, China; 4 University of Chinese Academy of Sciences, Beijing 100049, China; 5 College of Biological Sciences, University of California, Davis, One Shields Avenue, Davis, CA 95616, USA; 6 Center for Evolution and Conservation Biology, Southern Marine Science and Engineering Guangdong Laboratory, Guangzhou 511458, China

**Keywords:** venom peptide diversity, cysteine-knot peptide, anticoagulant therapy, activated protein C, thrombosis models

## Abstract

The development of effective anticoagulants remains a critical need in modern medicine, particularly for preventing and treating thromboembolic disorders, such as arterial thrombosis and deep vein thrombosis (DVT), as well as complications like ischemic stroke. This study identifies a cysteine-knotted peptide GC38 (sequence: GCSGKGARCAPSKCCSGLSCGRHGGNMYKSCEWNWKTG) derived from the venom gland transcriptome of the *Macrothele* sp. spider, which exerts thrombus-inhibitory effects by potentiating activated protein C (APC) activity. In vitro assays reveal that GC38 enhances APC activity, prolongs plasma clotting time, and shows no significant cytotoxicity or hemolytic activity. Mechanistically, GC38 interacts allosterically with APC; biolayer interferometry (BLI) confirms this direct interaction, with a dissociation constant *K*_D_ of 6.16 μM. Additionally, three in vivo thrombosis models (FeCl_3_-induced arterial occlusion, stasis-induced DVT, and cortical photothrombotic stroke) consistently demonstrated that GC38 was effective in alleviating thrombus formation, with tail-bleeding assays confirming its low hemorrhagic risk. Collectively, our findings position GC38 as a pioneering spider venom-derived lead molecule that addresses dual arterial and venous antithrombotic actions. This opens new avenues for developing spider venom-derived peptides as therapeutic agents targeting intravascular coagulation in arteries and veins.

## 1. Introduction

Thrombotic disorders driven by venous and arterial thrombosis, including arterial thrombosis, deep vein thrombosis, and their thromboembolic complications, such as ischemic stroke, are major contributors to global morbidity and mortality, with prevalence increasing significantly with age [1,2]. Anticoagulants are standard therapy for venous thromboembolism and arterial thrombosis-mediated disorders, particularly in acute settings [3]. However, current anticoagulants, such as warfarin and heparin, are often associated with significant bleeding risks and require close monitoring, which limits their clinical utility [4,5]. Given the high mortality rates and economic burden related to thrombosis [1,6], current prevention remains inadequate, highlighting the urgent need for novel antithrombotic therapies with improved safety profiles and novel mechanisms of action. Emerging anticoagulant peptides demonstrate promising advantages [7,8,9], including rapid onset of action and reduced bleeding risk compared to traditional anticoagulants. Clinically established examples include hirudin, a 65-amino acid thrombin inhibitor derived from medicinal leech—*Hirudo medicinalis*, which has been used in heparin-induced thrombocytopenia (HIT) management with lower bleeding risk than conventional therapies [10]. Another synthetic peptide, bivalirudin, shows superior safety profiles in percutaneous coronary intervention (PCI) with a reduction in major bleeding events compared to heparin [11]. Therefore, developing anticoagulants with novel mechanisms of action that can control thrombosis while minimizing bleeding risks represents a critical research priority.

The protein C pathway serves as a crucial endogenous anticoagulant system that tightly regulates blood coagulation [12,13]. Both protein C and its activated form (APC) share a conserved domain architecture: a Gla domain, followed by two EGF-like modules, and a C-terminal serine protease domain. Upon vascular injury, thrombin in complex with thrombomodulin and endothelial cell PC receptor activates circulating PC to generate APC—a serine protease that cleaves coagulation factors Va and VIIIa, thereby suppressing thrombin generation [12,14]. APC acts as both a key physiological anticoagulant and a promising therapeutic target [15]. This has spurred the development of safer APC modulators with optimized pharmacological profiles, including proteins that increase endogenous APC activity [16,17], and APC-mimetic peptides that replicate anticoagulant function for therapeutic use [18,19]. However, no peptide that directly potentiates endogenous APC activity has been reported to date.

Spider venom constitutes a vast natural library of secretory peptides that exhibit novel bioactivities and target-specific biological functions [20,21]. Although 53,325 spider species are reported worldwide (World Spider Catalog, https://wsc.nmbe.ch, accessed on 14 September 2025), only a small number of bioactive peptides derived from spider venom have been intensively studied [22]. Recent studies on *Macrothele* spider venoms reveal that *Macrothele yani* harbors the most extensively characterized venom gene repertoire. Its venom is predominantly composed of inhibitor cystine knot (ICK) peptides, with additional toxin families including proteases and other enzymatic components [23,24,25]. Yang et al. [24] reported the toxin diversity of the *Macrothele bannaensis* via integrated transcriptomic and proteomic analyses, further confirming the toxin diversity of *Macrothele* spider venoms.

Studies have focused on the use of venom peptides for anticoagulation therapy [26,27]. Spider venoms are rich sources of bioactive peptides with diverse pharmacological properties, including anticoagulant activity [28]. These peptides typically target key components of the coagulation cascade with high specificity, potency, and selectivity. For instance, several spider venom-derived peptides, such as LCTX-F2, have been identified as potentiators that enhance the activity of coagulation factors, including factor XIIa and thrombin [29], providing promising leads for developing hemostatic regulators. Conversely, the venom of *Eresus niger* contains phospholipase A_2_ with anticoagulant activity [30]. Collectively, spider venoms provide a source of both anticoagulant and procoagulant components, highlighting their potential for identifying novel modulators of coagulation. However, no anticoagulant peptides derived from spider venom have been identified to date.

In this study, we identified a novel anticoagulant peptide, GC38, from the venom of *Macrothele* sp. (Mygalomorphae: Macrothelidae) collected from Yunnan Province, China, via an integrated approach of transcriptomic screening and experimental validation. GC38 exerts potent anticoagulant activity by increasing APC activity. In vitro and in vivo assays confirmed that GC38 significantly prolongs plasma clotting time, attenuates thrombus formation, and maintains a safety profile regarding bleeding. These findings highlight GC38 as a promising candidate for developing novel anticoagulants.

## 2. Results

### 2.1. Identification and Characterization of the ICK Peptide GC38 from Macrothele sp. Venom

#### 2.1.1. In Virtual Screening of GC38 Binding to the Anticoagulant Target APC

**Figure 1:**
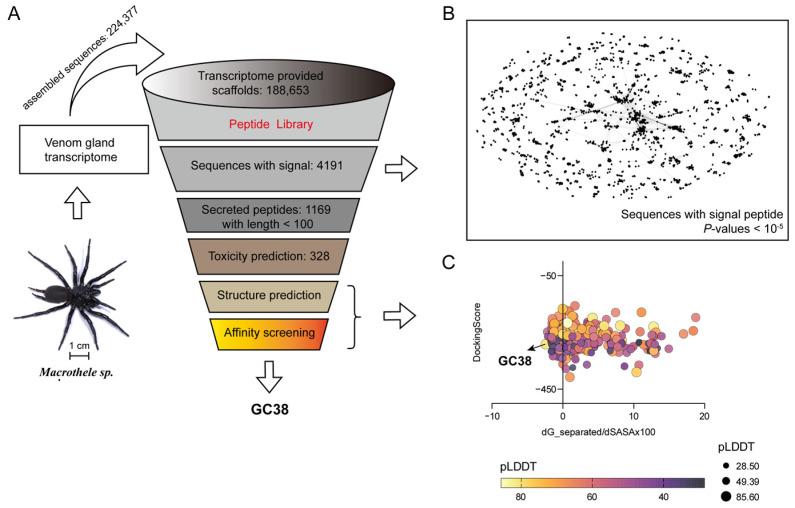
Pipeline for peptide GC38 identification and synthesis: (**A**) The multi-step virtual screening workflow of peptide GC38 targeting APC. (**B**) Sequence similarity network of transcripts from the de novo assembled transcriptome with signal peptides. Dots represent individual sequences. (**C**) Peptide screening by structural stability (Rosetta interface score: dG_separated/dSASA × 100) vs. binding affinity (DockingScore) with structure confidence mapping (pLDDT, predicted local distance difference test).

To identify potential anticoagulant peptides from the *Macrothele* sp. venom gland transcriptome, we performed a stepwise virtual screening workflow (Figure 1A). First, total RNA from *Macrothele* sp. venom glands was subjected to sequence assembly, yielding 224,377 unique assembled sequences, which were then translated into 188,653 protein sequences. Next, precursor sequences were annotated for secretion signals, retaining 4191 sequences with valid signal peptides.

Subsequently, the 4191 secreted precursor sequences were processed for clustering and grouping. A sequence similarity network was constructed using the CLANS tool (E-value < 10^−5^), which revealed extensive diversity in *Macrothele* sp. venom components (Figure 1B). These precursors that followed were grouped via BLAST v2.9.0+ searches against each other. Singleton sequences with no similar sequences in the library were omitted. Only a few sequences were clustered closely, and there were many groups.

After identifying secreted peptides, we truncated the sequences to 100 amino acids, and finally obtained 1169 peptide sequences. For identifying anticoagulant peptides, 328 sequences were retained after excluding 841 sequences predicted to have toxic or hemolytic activity. Then, these 328 peptides were structurally predicted and docked to APC, with binding affinity evaluated via a comprehensive comparison of HDOCK Docking-Score and Rosetta Interface scores (dG_separated/dSASA × 100). GC38 was characterized by its robust binding to APC and a conserved structure with the highest pLDDT value, indicating structural reliability (Figure 1C). Subsequently, GC38 was subjected to BLAST searches against the NR database (threshold E < 0.001), with no matching sequences identified, confirming its sequence novelty. Based on its favorable binding to APC, structural stability, non-toxic profile, and sequence novelty, GC38 was selected as the lead peptide for subsequent experimental validation.

#### 2.1.2. Identification of GC38 from Spider Venom Gland Transcriptome

**Figure 2:**
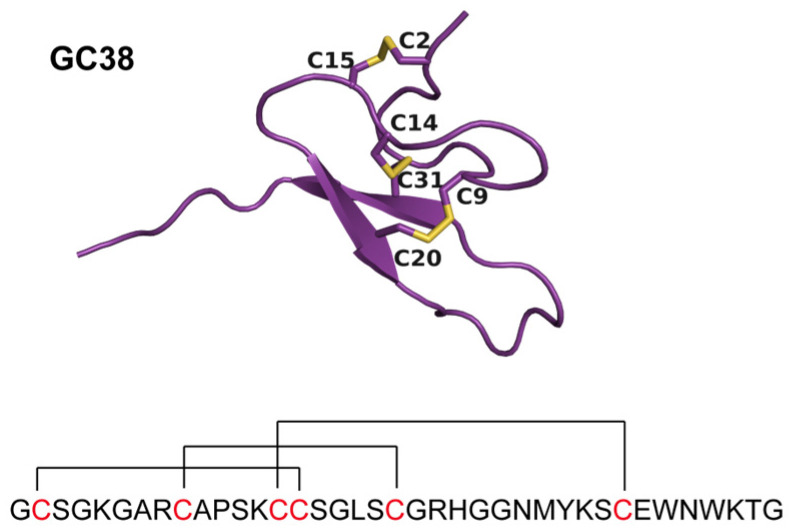
The sequence and predicted structure of GC38. Disulfide bonds in the predicted structure are formed between Cys2 and Cys15, Cys9 and Cys20, and Cys14 and Cys31 (highlighted in yellow sticks).

From venom gland-specific transcripts of *Macrothele* sp. venom, a candidate sequence encoding a cysteine-rich peptide named GC38 was identified. GC corresponds to the first two amino acids of the peptide sequence, and 38 refers to the 38 amino acids in the mature form of GC38. GC38 is predicted to consist of six conserved cysteines, forming three disulfide bridges. AlphaFold2 predicted the structure of GC38 with a pLDDT of 80.8. By structure modeling, the disulfide formation was mapped as Cys^2^-Cys^15^, Cys^9^-Cys^20^, and Cys^14^-Cys^31^, indicating an inhibitor cystine knot (ICK) fold structure (Figure 2).

#### 2.1.3. Synthesis and Purification of Peptide GC38 

Disulfide-bonded GC38 was obtained by oxidative refolding, with a yield of 28.5%. HPLC results showed the retention time of 16.5 min for refolded GC38 (Figure S1), with a purity of >95% for GC38 synthesis. By electrospray ionization mass spectrometry, the molecular weight (MW) of disulfide-bonded GC38 (theoretical: 3962.45 Da, observed: 3963.70 Da) validates the synthetic results that form three disulfides (Figure S2).

### 2.2. The Effect of GC38 Peptide on APC and Its In Vitro Anticoagulant Activity

**Figure 3:**
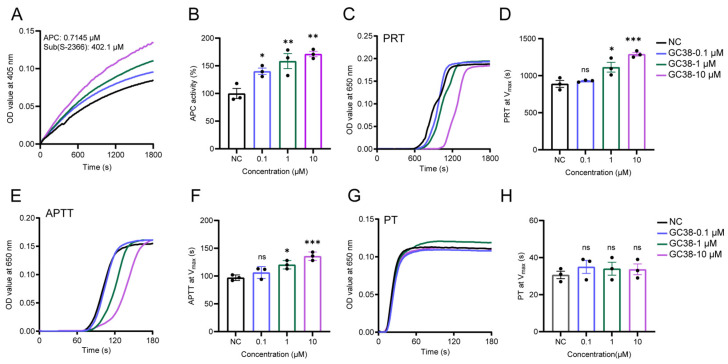
GC38 enhances APC and prolongs the clotting time: (**A**) Enzyme kinetic analysis of GC38 on APC (0.7145 μM) using substrate S-2366 (402.1 μM). (**B**) The residual activity of APC at V_max_. (**C**) The plasma recalcification time (PRT) curves of PBS and GC38 at 0.1–10 μM concentrations. (**D**) Time points at PRT reached V_max_. (**E**) The activated partial thromboplastin time (APTT) curves of PBS and GC38 at 0.1–10 μM concentrations. (**F**) Time points at APTT reached V_max_. (**G**) The prothrombin time (PT) curves of PBS and GC38 at 0.1–10 μM concentrations. (**H**) Time points at PT reached V_max_. (**A**,**C**,**E**,**G**) The curves are the mean of three independent measurements. (**B**,**D**,**F**,**H**) Data are presented as the mean ± standard error of the mean (SEM) (n = 3); * *p* < 0.05, ** *p* < 0.01, *** *p* < 0.001 versus the PBS control group, and ns means no significant differences.

To investigate whether the anticoagulant mechanism of GC38 involves increasing the enzymatic activity of APC, this study assessed the regulatory effect of GC38 on APC’s enzymatic activity in vivo through enzyme kinetics and coagulation assays. GC38 enhanced APC activity in a concentration-dependent manner from 0.1 to 10 μM (Figure 3A). At 10 μM concentration, the activity of APC was increased to 171% at maximum catalytic velocity (V_max_) (Figure 3B). Plasma recalcification time (PRT) assays demonstrated that GC38 prolonged clotting time in plasma in a dose-responsive manner (Figure 3C). The clotting time required for PRT to reach V_max_ is shown in Figure 3D. The clotting time for GC38 at a concentration of 10 μM was 1.4-fold compared to the negative control group. Importantly, GC38 significantly extended the activated partial thromboplastin time (APTT) at 10 μM (1.4-fold vs. control, Figure 3E,F) but exhibited no apparent effects on prothrombin time (PT) (Figure 3G,H).

### 2.3. The Potential Binding Mechanism of GC38 and APC

#### 2.3.1. Direct Binding of GC38 to APC

**Figure 4:**
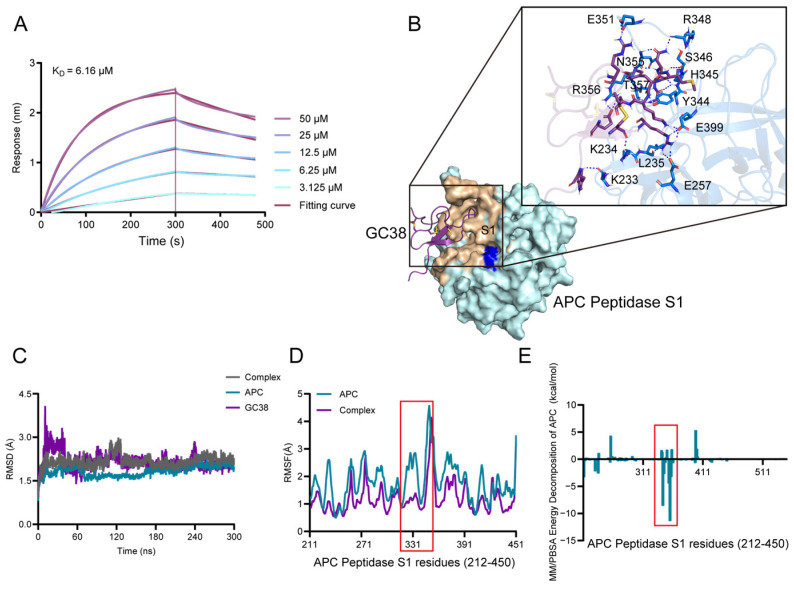
The proposed binding mechanism of GC38: (**A**) The biolayer interferometry (BLI) curves of GC38 binding to APC at concentrations of 3.125–50 μM. (**B**) Overall docking pose of GC38-APC. Local magnification reveals residues of APC that formed key hydrogen bonds at the binding cleft. The key residues involved in the interaction were labeled, and the blue dashed lines represent hydrogen bonding interactions. (**C**) The RMSD curves of GC38, APC, and GC38-APC complex backbones during 300 ns simulations. (**D**) The RMSF curves of the APC and GC38-APC complex. The most flexible region was highlighted in a red box. (**E**) Per-residue MM/PBSA binding energy decomposition of the APC contribution by residues. The region that contributed most to binding was highlighted in a red box.

Biolayer interferometry (BLI) was employed to assess the binding kinetics and affinity between GC38 and activated protein C (APC) [31]. BLI analysis confirmed direct binding of GC38 to APC (Figure 4A) with a dissociation constant (*K*_D_) of 6.16 μM. Complementary to enzyme kinetic assays, GC38 potentiated APC activity in a dose-dependent manner, indicating functional enhancement of anticoagulant activity through targeted APC engagement.

#### 2.3.2. Molecular Docking and Binding Energy Analysis from MD Simulations

The results of GC38 docking with APC are shown in Figure 4B. Rosetta docking predicted the GC38-APC complex structure, identifying key interactions [32]. Hydrogen bonding predominantly stabilized the complex. The bound amino acids of APC are LYS233, LYS234, LEU235, GLU257, TYR344, HIS345, SER346, ARG348, GLU351, ASN355, ARG356, THR357, and GLU399. It is inferred that the binding site of GC38 to APC was the exosite of APC [32,33]. APC exosite formed 18 hydrogen bonds with GC38. GC38 was combined with the exosite of APC by conventional hydrogen bonds via mainly residues 20 to 30, which is the core of the cysteine-knotted structure. Additionally, the wheat color shows the contact within 0.4 nm, near the catalysis center (blue color). Although GC38 did not directly bind to the catalytic triad of APC, it combined with the surroundings of the triad, suggesting an allosteric mechanism. 300 ns molecular dynamics simulations confirmed the system reached equilibration (Figure 4C). Root mean square fluctuation (RMSF) analysis revealed enhanced flexibility in APC’s exosite during simulations (Figure 4D), consistent with its role in heparin binding. The calculated MM/PBSA binding energy between APC and GC38 was −96.5594 kcal/mol. Energy decomposition implicated exosite residues (ΔG > −3.0 kcal/mol) as major contributors to binding (Figure 4E). Importantly, the binding of GC38 to APC induced a conformational shift, potentially increasing anticoagulant activity through allosteric modulation of APC.

### 2.4. Cytotoxicity and Hemolysis Profiles of GC38

**Figure 5:**
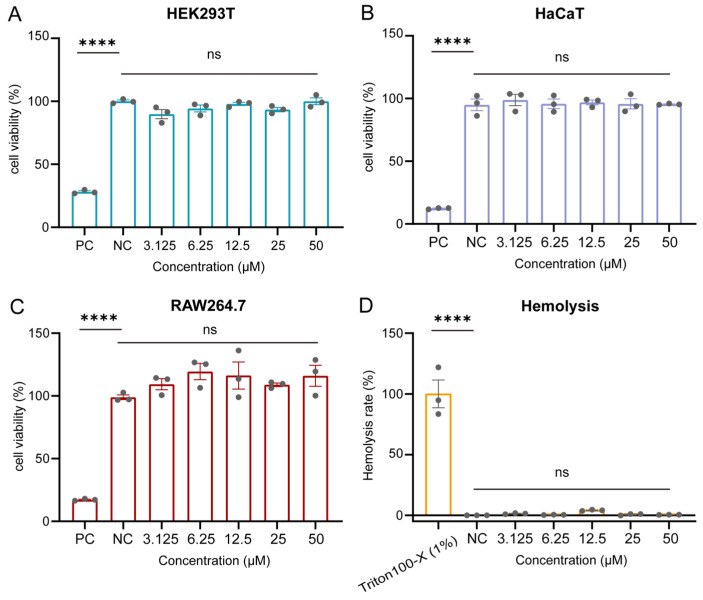
In vitro safety evaluation of GC38: (**A**) Cytotoxicity of GC38 on HEK293T cells. (**B**) Cytotoxicity of GC38 on HaCaT cells. (**C**) Cytotoxicity of GC38 on RAW264.7 cells. (**D**) Hemolytic effects of GC38 on mouse red blood cells. GC38 concentration: 3.125–50 μM. All data are presented as the mean ± SEM (n = 3); **** *p* < 0.0001 versus the PBS control group, and ns means no significant differences.

Before conducting mouse model studies, the in vitro cytotoxicity and hemolytic potential of GC38 were evaluated. GC38 exhibited negligible cytotoxicity in HEK293T, RAW264.7, and HaCaT cell lines across the concentration range (0.1–50 μM), with cell viability greater than 95% at 50 μM after 24 h of exposure (Figure 5A–C). Consistently, its hemolytic activity remained below 3% at 50 μM compared to a 1% Triton X-100 positive control (Figure 5D). These results are consistent with toxicity predictions, indicating that peptide GC38 is not toxic per se and is safe for in vivo testing.

### 2.5. In Vivo Efficacy of the GC38 Peptide as an Antithrombotic, Anticoagulant, and Bleeding Treatment

The in vivo anticoagulation effects of GC38 were evaluated in three mouse models: FeCl_3_-induced carotid artery thrombosis, stasis-induced deep vein thrombosis (DVT), and a cortical photothrombotic stroke (CPS) model. These well-established models were employed to assess the efficacy of GC38 across distinct thrombotic conditions—arterial occlusion, venous thrombosis, and cerebral ischemia—providing comprehensive coverage of clinically relevant thrombosis types.

#### 2.5.1. Antithrombotic and Anticoagulant Effects of GC38 in FeCl_3_-Induced Carotid Artery Thrombosis Model

**Figure 6:**
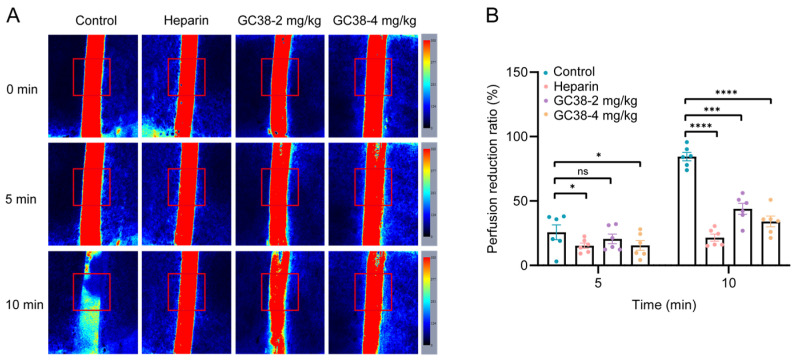
Effect of GC38 on FeCl_3_-induced carotid artery thrombosis in mice: (**A**) Representative images showing relative blood flow intensity in the injured carotid artery at the indicated times after topical application of 10% FeCl_3_. The region of interest (ROI) for blood flow recording was in the red box. The color bar on the right indicates relative blood flow intensity, with red representing high flow and blue representing low or no flow. (**B**) Blood flow reduction ratio in mice (n = 6 per group) treated with PBS (control), GC38 (2 or 4 mg/kg), or heparin (heparin sodium, 1000 U/kg). Data are presented as the mean ± SEM; * *p* < 0.05, *** *p* < 0.001, **** *p* < 0.0001 compared with the PBS control group, and ns means no significant differences.

Topical application of 10% FeCl_3_ to surgically exposed carotid arteries induced rapid thrombus formation in mice. Also, mice were pretreated with GC38 at 2 and 4 mg/kg (*i.v.*) for 10 min before FeCl_3_ application. At 5 min, GC38 at doses of 2 mg/kg and 4 mg/kg significantly reduced blood flow by 20.6% and 11.8%, respectively. At 10 min, the blood flow was reduced by 44.0% and 29.4%. As measured by Doppler flowmetry, GC38 alleviated FeCl_3_-induced vascular occlusion in mice (Figure 6A,B).

#### 2.5.2. Antithrombotic and Anticoagulant Efficacy of GC38 in Stasis-Induced Deep Vein Thrombosis (DVT) Model

**Figure 7:**
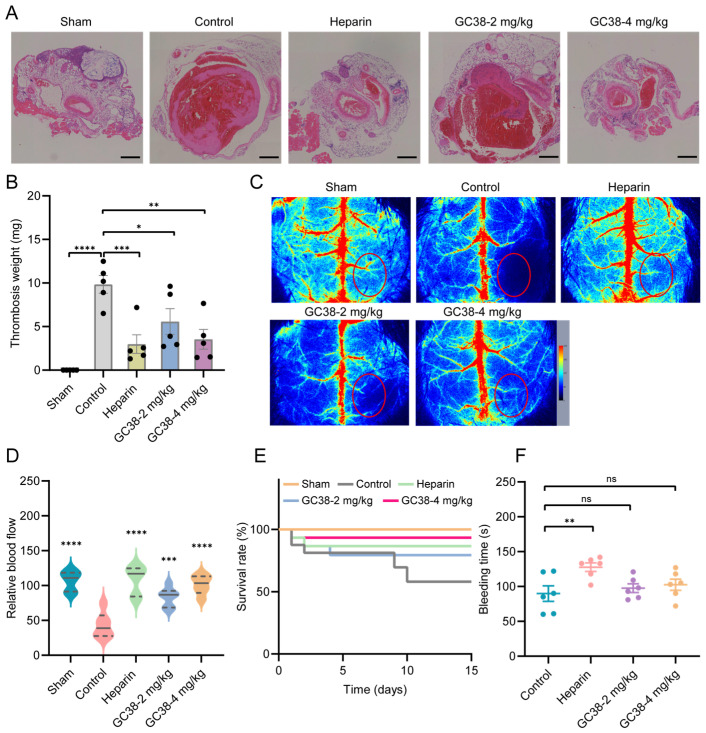
Antithrombotic efficacy of GC38 in murine stasis-induced deep vein thrombosis (DVT), cortical photothrombotic stroke (CPS) models, and bleeding risk of GC38: (**A**) Representative hematoxylin and eosin (HE)-stained histopathological sections of the inferior vena cava with thrombi in the mouse DVT model. Scale bar, 200 μm. (**B**) The wet weight of thrombi in the control (PBS), GC38 (2 or 4 mg/kg), and heparin (heparin sodium, 1000 U/kg) groups (n = 5). (**C**) The representative pictures of the CPS model treated with PBS, GC38 (2 and 4 mg/kg), and heparin (heparin sodium, 1000 U/kg). The red circle represents photothrombosis-inducing regions of the CPS mouse model. (**D**) The blood flow recording in the region of interest (ROI, red circled area) was monitored by laser speckle perfusion (LSP) imaging (n = 6). (**E**) The survival rate in the mouse CPS model was recorded for 15 consecutive days (n = 6). (**F**) The tail bleeding time of GC38. PBS, GC38 (2 or 4 mg/kg), and heparin (heparin sodium, 1000 U/kg) were injected intravenously (n = 6). The Sham group underwent identical surgical procedures except for thrombosis induction, serving as a control. Data are mean ± SEM; * *p* < 0.05, ** *p* < 0.01, *** *p* < 0.001, **** *p* < 0.0001 compared with PBS control group, and ns means no significant differences.

The antithrombotic effect of GC38 was evaluated in a mouse model of deep DVT. Following 24 h of inferior vena cava (IVC) ligation, control mice developed overt thrombi in the IVC (Figure 7A). GC38 treatment (4 mg/kg, *i.v.*) significantly reduced thrombus wet weight by 64.0% compared to vehicle control (n = 5 per group), indicating reduced thrombus burden and suppressed formation. Histological analysis further confirmed a decrease in thrombus area in GC38-treated mice (Figure 7B). These data demonstrate that GC38 effectively suppresses venous thrombus formation, supporting its potential for treating thrombus formation in DVT.

#### 2.5.3. Antithrombotic and Anticoagulant Efficacy of GC38 in Cortical Photothrombotic Stroke (CPS) Model

In the mouse CPS model, ischemic lesions were quantified using the LPS imaging system, which measures blood perfusion and directly reflects the severity of ischemia (n = 6 per group). At a dose of 4 mg/kg, GC38 delayed microvascular occlusion and alleviated the formation of ischemic lesions (Figure 7C). Real-time blood perfusion monitoring revealed that GC38 treatment (2 and 4 mg/kg) significantly attenuated blood flow reduction, with the extent of decrease limited to only 22.0% and 6.4%, respectively. In contrast, the vehicle control group exhibited a 40.1% reduction in blood flow (Figure 7D). Furthermore, GC38 improved the 15-day survival rate of mice compared to the untreated group, indicating its sustained neuroprotective effects (Figure 7E).

#### 2.5.4. Bleeding Risk Assessment

Tail bleeding times in GC38-treated mice (2 mg/kg: 97.6 s; 4 mg/kg: 100.6 s) were comparable to the vehicle control group (89.8 s, n = 6 per group, Figure 7F). No increased bleeding risk was observed between the vehicle control group and the GC38 groups. In contrast, heparin sodium (1000 U/kg) significantly prolonged bleeding to 127.6 s. Compared to heparin sodium, GC38 shows an improved safety profile. Combined with its potent antithrombotic efficacy, these findings confirm that GC38 exhibits minimal bleeding risk in vivo. Collectively, these data establish GC38 as a promising anticoagulant candidate with negligible cytotoxicity, low hemolytic activity, and reduced hemorrhagic complications.

## 3. Discussion

Natural sources have long been recognized as a reservoir of anticoagulant bioactive substances [34,35]. Peptides derived from spider venom could be a novel source of anticoagulants. Spiders, as ancient arthropod animals, have evolved a diverse array of bioactive peptides targeting distinct biological activities over more than 300 million years of venom system evolution [36]. These venom peptides not only exhibit rich structural folding patterns (e.g., the inhibitor cystine knot motif and intrinsically disordered regions) that form a crucial molecular repository for drug discovery, but also demonstrate remarkable chemical stability, particularly for disulfide-rich variants [37]. When such structurally stable peptides concurrently possess pharmacological activity, their druggability is significantly enhanced.

In this study, from the venom gland transcriptome of *Macrothele* sp., we identified and characterized GC38, a potent anticoagulant targeting APC. GC38 was predicted to adopt an inhibitory cystine knot (ICK) scaffold with anticoagulation activity. Our work demonstrates that ICK motifs represent a novel molecular template for designing anticoagulant peptides. This structural framework exhibits three key advantages over traditional scaffolds: Enhanced stability from three conserved disulfide bonds (CysI-CysIV, CysII-CysV, and CysIII-CysVI) forming the characteristic knot topology [38].

To the best of our knowledge, GC38 is the first ICK-type peptide identified from the venom gland transcriptome of Macrothelidae to increase APC activity. This structural feature not only enhances binding stability but also exhibits intriguing bioactivity worthy of further investigation. The presence of anticoagulant components in spider venom is an evolutionary product shaped by predation strategies and survival competition, involving molecular adaptive evolution under multiple ecological pressures.

The exceptionally expanded sequence space of the *Macrothele* sp. spider transcriptome represents a transformative strategic resource for peptide drug discovery. This biological treasure trove transcends conventional libraries by offering unparalleled structural and functional diversity-epitomized by the anticoagulant peptide GC38, which inhibits thrombosis with structural novelty that emerges from conserved disulfide frameworks honed to target biological sites on critical targets like APC. This underscores the importance of venom biodiversity not merely as a source of leads, but as a blueprint for biologically inspired design, and will propel peptide therapeutics into an era of precision targeting for intractable disorders.

APC is a critical serine protease in the coagulation system and can be a target for antithrombotics. Based on the results showing that GC38 increases APC activity, GC38 likely binds to the non-catalytic domain of APC, stabilizing its active conformation. In PRT experiments, the dose-dependent prolongation of coagulation time suggests that GC38 possesses a controllable anticoagulation window, making it suitable for long-term antithrombotic therapy. APTT and PT are demonstrated to be associated with the intrinsic coagulation pathway, potentially targeting the APC–protein S complex, and increasing APC’s inactivation efficiency of factors Va/VIIIa, thereby inhibiting the critical amplification step of thrombin generation. We reported a mechanism showing that GC38 potentiates the enzymatic activity of APC by combining the key regions of APC. Here, the docking model showed that GC38 interacted with the exosite region of APC. This provides opportunities for drug development targeting mature functional sites, providing a new direction for the application of spider-derived peptides with antithrombotic activity.

Moreover, although some small-molecule drugs have been used for anticoagulation treatment, bleeding remains a major concern in anticoagulant therapy [39]. Using the widely validated mouse tail bleeding assay, we found that heparin sodium consistently prolonged bleeding time compared to GC38 across experimental conditions. These results demonstrate GC38’s substantially lower bleeding risk than clinical heparin, highlighting its improved safety profile.

In total, we leveraged transcriptomic resources from naturally venomous organisms to capture sequence space diversity, establishing a systematic peptide screening pipeline. We identified the first anticoagulant cysteine-knotted peptide GC38 and demonstrated dual anticoagulant and thrombolytic activities with low bleeding risk, achieving pan-vascular targeting (both arterial and venous systems). However, several limitations of this study should be noted. First, experimental validation for the predicted ICK structure of GC38 is currently lacking. The classification of GC38 as an ICK-type peptide was solely based on in silico sequence analysis and structural prediction rather than experimental evidence such as nuclear magnetic resonance (NMR) spectroscopy. Second, the structure–activity analysis proposed for the potential mechanism requires further experimental validation to confirm the critical functional sites responsible for anticoagulant activity. Additionally, the thrombosis model does not fully recapitulate the complex pathophysiology of human ischemic stroke. In future studies, we will employ peptidomics and proteomics approaches to systematically explore the composition of *Macrothele* spider venom. Furthermore, the structure and the underlying mechanisms will be elucidated through experimental verification, and the therapeutic potential of GC38 will be evaluated in a mouse model of ischemic stroke.

## 4. Materials and Methods

### 4.1. Chemicals and Materials

Human Activated Protein C (APC-1.0) was purchased from Enzyme Research Laboratories, Inc., South Bend, IN, USA. Chromogenix substrate S-2366: pyroGlu-Pro-Arg-pNA (S-2366, Km = 0.2 mM, kcat = 190 s^−1^, CAS: S821090) was obtained from Diapharma Group, Inc., West Chester, OH, USA. Activated Partial Thromboplastin Time (APTT) Test Kit (TC0306) was bought from Senkey Biotechnology Co., Ltd., Kunming, China. Prothrombin Time (PT) Test Kit (GMS10176) was purchased from GENMED Scientifics Inc., Shanghai, China. Streptavidin (SA) Biosensors (18-5019) were obtained from Sartorius AG, Göttingen, Lower Saxony, Germany. PBS (21-040-CVC) was bought from Corning Incorporated, Corning, NY, USA. Tween-20 (BS361B) was purchased from Biosharp Life Sciences, Hefei, Anhui, China. Trifluoroacetic acid (TFA, T818778-500ml, CAS: 76-05-1) and CaCl_2_ (C805225) were obtained from Macklin Biochemical Co., Ltd., Shanghai, China. L-oxidized glutathione (GSSG, V900363), Rose Bengal Dye (content 95%, CAS: 632-69-9, 330000-5G), and HEPES (V900477-500G) were purchased from Sigma-Aldrich LLC, St. Louis, MO, USA (Shanghai branch: Sigma-Aldrich Shanghai Trading Co., Ltd., Shanghai, China). A reversed-phase C18 column (Waters, XBridge BEH, 130 Å, 2.5 µm, 4.6 mm × 150 mm) was used for high-performance liquid chromatography (HPLC), which was purchased from Waters Corporation, Milford, MA, USA. Glycine (HY-Y0966), Biotin-PEG12-NHS ester (HY-140892), and Cell Counting Kit-8 (CCK-8, HY-K0301) were bought from MedChemExpress LLC, Monmouth Junction, NJ, USA (Shanghai branch: MedChemExpress Shanghai Co., Ltd., Shanghai, China). DMEM (10-013-CVRC) and RPMI 1640 (10-040-CVRC) were purchased from Corning Incorporated, Corning, NY, USA. Trypsin-EDTA (0.25%), phenol red (25200056), and FBS (10099141C) were obtained from Gibco Life Technologies, Grand Island, NY, USA. Heparin Sodium (1170GR005) was purchased from Biofroxx GmbH, Marie-Curie-Str. 3 Einhausen, Germany. All other chemicals utilized in this study were of analytical grade.

### 4.2. Identification and Characterization of the Peptide GC38 from Macrothele sp. Venom

#### 4.2.1. Spider Collection, Venom Gland Dissection, and Venom Gland Transcriptome Sequencing and Assembly

*Macrothele* sp. spiders were collected from the *Gaoligong* Mountains, Yunnan Province, China. Their venom glands were aseptically dissected on ice, immediately snap-frozen, and stored in liquid nitrogen. Subsequently, the samples were sent to Novogene (Beijing, China) for total RNA extraction and subsequent sequencing. Total RNA was extracted from the venom gland of *Macrothele* sp. using the TRIzol reagent (ThermoFisher Scientific, Waltham, MA, USA), followed by mRNA enrichment and cDNA library construction. Next-generation sequencing (NGS) was carried out on the Illumina NovaSeq 6000 platform, generating paired-end reads. Raw sequencing data were first quality-checked using FastQC v0.11.9, and low-quality reads and adapters were then removed using fastp v0.23.4. De novo transcriptome assembly was performed using RNA-Bloom v1.4.3 [40] with default parameters. Finally, the assembled transcripts were translated into protein sequences using Transdecoder v5.5.0.

#### 4.2.2. Sequence Similarity Network Construction

Protein sequences with signal peptides were clustered using CLANS software (v29.05.2012) [41], with clustering based on a BLASTp E-value threshold of 1 × 10^−5^. Clustering was run with 20,000 iterations, and the clustered sequences were visualized as a sequence similarity network. In this network, connections between sequences were shown only when their BLASTp E-value met the aforementioned 1 × 10^−5^ threshold.

#### 4.2.3. GC38 Identification Based on Virtual Screening

The SignalP 6.0 software [42] was used to predict signal peptides, and TMHMM v2.0 [43] was used to predict transmembrane helices, with secretory peptides identified as those lacking transmembrane domains. Translated protein sequences were annotated using BLASTp (E-value ≤ 1 × 10^−5^) against the UniProtKB/Swiss-Prot database. Additionally, to focus on small bioactive peptides, sequences were filtered to retain those with lengths < 100 residues. Three-dimensional (3D) structures of all sequences were predicted using AlphaFold2 [44], with each model assigned a pLDDT score (predictive local distance difference test, a metric for assessing model confidence). ToxPred and HemoPI2 [45] were used to predict peptide toxicity and hemolytic activity, respectively. Peptides with low predicted toxicity and no predicted hemolysis were retained. HDOCK [46] was used to perform molecular docking to investigate the binding affinity between the peptides and APC. The crystal structure of APC (PDB ID: 1AUT) was retrieved from the Protein Data Bank (PDB) [47]. The protein structure was preprocessed using PyMOL v3.1 software (https://pymol.org/) to remove water molecules and heteroatoms. Hydrogen atoms were subsequently added, and the structure was energy-minimized using molecular dynamic software package AMBER20 (University of California, San Francisco, CA, USA) to ensure structural stability. Rosetta InterfaceAnalyzer was used to further analyze peptide–APC binding interactions, with results compared between HDOCK scores and Rosetta interface scores (dG_separated/dSASA × 100). The selected peptides were based on sequence novelty, safety, and affinity [48].

#### 4.2.4. Chemical Synthesis of GC38

The linear form of GC38 was obtained from GO TOP PEPTIDE BIOTECH Co., Ltd. (Hangzhou, China) using 9-fluorenylmethoxycarbonyl (Fmoc)-based solid-phase peptide synthesis (SPPS). Fmoc-Gly-Wang Resin (loading = 0.29 mmol/g) was used as the solid support for SPPS. After cleavage from the resin using a TFA-based cocktail and precipitation in cold diethyl ether. For oxidative folding, the purified linear peptide was dissolved at 0.5 mg/mL in a buffer containing 3 mM oxidized glutathione (GSSG) and 0.1 M glycine-NaOH (pH 8.5), and the solution was then incubated at 25 °C for 24 h [49]. The disulfide-bonded peptide GC38 was purified by RP-HPLC on a C18 column (Waters XBridge BEH, 130 Å, 2.5 μm, 4.6 × 150 mm) with a linear gradient of 5–45% acetonitrile (in 0.1% aqueous TFA) at a flow rate of 1.0 mL/min over 40 min. The molecular mass of GC38 was confirmed by electrospray ionization mass spectrometry. Finally, the purified GC38 was lyophilized under vacuum and stored at −80 °C until use.

### 4.3. In Vitro Assays for Evaluating GC38’s Effects on Coagulation

#### 4.3.1. Measurement of APC Activity by Chromogenic Substrate Assay

APC (0.7145 μM) was pre-incubated with varying concentrations of GC38 (0.1, 1 and 10 μM) in PBS at 37 °C for 10 min. Reactions were initiated by adding S-2366 to a final concentration of 402.1 μM (final volume: 100 μL). Kinetic measurements were performed at 37 °C using a Multi-Mode Microplate Reader (BioTek Synergy™ H1, Winooski, VT, USA) with absorbance at 405 nm recorded every 10 s for 30 min. The control group contained APC and S-2366 with PBS. All experiments were conducted in triplicate, and data are presented as mean ± SEM.

#### 4.3.2. Plasma Recalcification Assay

Plasma recalcification time (PRT) was used to evaluate the effects of GC38 on the coagulation pathway. Fresh plasma was incubated with GC38 (0.1, 1, and 10 µM) for 10 min at 37 °C. After incubation, the plasma was mixed with an equal volume of 25 mM calcium chloride solution, and the timer was immediately started. The time required for plasma clot formation was recorded. The group treated with PBS served as the control. Each concentration was tested in triplicate, and the average value and PRT at maximum reactive velocity were reported.

#### 4.3.3. Activated Partial Thromboplastin Time (APTT) and Prothrombin Time (PT)

APTT and PT were measured with commercially available kits according to the manufacturer’s instructions [50]. For APTT determination, 20 μL human plasma was pre-incubated with GC38 (0.1, 1, and 10 µM) at 37 °C for 5 min, and then 30 μL APTT reagent was added and incubated for another 5 min. Subsequently, 40 μL pre-warmed CaCl2 (37 °C) was added to initiate the intrinsic clotting cascade. The intensity of the scattered light was monitored until the signals reached saturation. For PT determination, 20 μL human plasma was pre-incubated with GC38 (0.1, 1, and 10 µM) at 37 °C for 5 min. Then, 75 μL PT reagent was added to initiate the extrinsic clotting cascade. The intensity of the scattered light was monitored until the signals reached saturation. The coagulation time (APTT or PT) was defined as the time point at which the scattered light signal reached the midpoint between the baseline and maximum amplitude. All concentrations were tested in triplicate. Results are shown as mean values, and the time at maximum reactive velocity was also reported.

### 4.4. Potential Anticoagulant Mechanism of GC38

#### 4.4.1. Bio-Layer Interferometry (BLI) Assay

The binding affinity between APC and GC38 was measured using the Octet^®^ R2 system (ForteBio, Fremont, CA, USA) with Streptavidin (SA) biosensors. APC was diluted with BLI binding buffer (PBS with 0.02% Tween-20) and biotinylated with Biotin-PEG12-NHS ester at room temperature for 1 h. Excess Biotin-PEG12-NHS ester was removed by ultrafiltration. Before testing, sensors were soaked in BLI binding buffer for at least 10 min. In addition, one buffer-only sensor was reserved as a control, and another APC-only sensor was set to determine the background influence. Then, the BLI run was performed with the following steps: a 60-s buffer-only baseline to equilibrate the sensors. Biotinylated APC at a fixed concentration of 10 µg/mL was immobilized onto biosensors for 120 s and balanced with the assay buffer for 60 s. The biosensors were then exposed to varying concentrations of GC38 (50, 25, 12.5, 6.25, and 3.125 μM) for 300 s, followed by a dissociation process in the same buffer for 180 s. Data were acquired and analyzed using Octet^®^ Data Acquisition and Analysis software version 8.2. For full kinetic measurements, the buffer-only baseline sample was used to subtract the background from all samples and fit both the association and dissociation curves together to calculate the equilibrium dissociation constant (*K*_D_) values. Each GC38 concentration test was repeated three times, and the average value was chosen as the result.

#### 4.4.2. Docking and Refining the Model of GC38 Binding to APC

To elucidate the binding mode of GC38 to APC, molecular docking simulations were conducted using Rosetta 3.13 [51,52]. The crystal structure of human APC (PDB ID: 1AUT) was prepared by removing water and unrecognized atoms. GC38 was constructed with the AlphaFold2 webserver [44], followed by energetic minimization. Global docking was performed via RosettaDock with default parameters, generating 10,000 decoy poses. The top-ranked poses by interface score underwent local refinement, allowing side-chain flexibility. The conformations with the lowest Rosetta Energy Unit (REU) values were selected for binding mode analysis.

#### 4.4.3. Molecular Dynamics Simulation of the GC38-APC Complex

MD simulations were performed with AMBER20 (University of California, San Francisco, CA, USA) to assess the stable structure of GC38-APC complex. The initial structure was derived from the top-ranked RosettaDock pose. APC and GC38 were modeled with the ff19SB force field. The system was solvated in a TIP3P water box, extending 10 Å from the solute in all directions, and neutralized by adding appropriate counterions (Na^+^ or Cl^−^). Energy minimization of the system was performed using the default parameters in three steps: first, the solvent and ions were minimized while restraining the solute with a harmonic potential (500 kcal·mol^−1^·Å^−2^). Second, the backbone of the solute was restrained. Third, the entire system was minimized without restraints. The system was gradually heated from 0 to 310 K over 500 ps under constant volume conditions, followed by equilibration at 310 K and 1 atm for 500 ps in the NPT ensemble. Production MD simulations were carried out for 300 ns with a time step of 2 fs, and coordinates were saved every 100 ps for subsequent analysis. Two systems (GC38-APC and APC alone) were subjected to molecular dynamics simulations. RMSD and RMSF were calculated to evaluate the simulation equilibration and structural flexibility. The binding interactions were quantified via hydrogen bonds (criteria: distance < 3.5 Å, angle > 120°) and hydrophobic contacts (mass center distance < 3.5 Å). These interactions were visualized and analyzed using PyMOL v3.1 software.

#### 4.4.4. Binding Affinity Quantification Through MM/PBSA and Energy Decomposition

The binding free energy (Δ*G*_bind_) of the GC38-APC complex was calculated using the molecular mechanics/Poisson–Boltzmann surface area (MM/PBSA) method implemented in AMBER20 (University of California, San Francisco, CA, USA). The Δ*G*_bind_ for each of the 500 snapshots extracted from the last 100 ns of the equilibrated MD trajectory was computed. Per-residue energy decomposition was also performed using AMBER20 to identify key binding hotspots.

### 4.5. In Vitro Safety Assessment of GC38: Cytotoxicity and Hemolysis

#### 4.5.1. Cytotoxicity Assay

The cytotoxicity of GC38 was evaluated in human embryonic kidney cells (HEK293T), human keratinocytes (HaCaT), and murine macrophage (RAW264.7) cell lines. The cells were from Kunming Cell Bank, Chinese Academy of Sciences. HEK293T and HaCaT cells were cultured in DMEM supplemented with 10% fetal bovine serum (FBS) and 1% penicillin-streptomycin, and RAW264.7 cells were maintained in RPMI 1640 containing 10% FBS and 1% penicillin–streptomycin, with all cell lines incubated at 37 °C under 5% CO_2_. For cytotoxicity assessment, cells were seeded at a density of 1 × 10^5^ cells per well in 96-well plates and incubated for 12 h. GC38 was serially diluted in the serum-free medium to concentrations ranging from 3.125 to 50 µM and added to the cells. After 24 h incubation, cell viability was determined using the CCK-8 assay (10 μL/well). Absorbance was measured at 450 nm using a multi-mode microplate reader (BioTek Synergy™ H1, Winooski, VT, USA). Untreated cells and cells treated with 10% DMSO served as negative and positive controls, respectively. Viability percentages were calculated relative to the untreated control. All experiments were conducted in triplicate, and data were expressed as mean ± SEM.

#### 4.5.2. Hemolysis Assay

The hemolytic activity of GC38 was assessed using mouse red blood cells (RBCs). Fresh human blood was centrifuged at 1500 rpm for 5 min to isolate RBCs, followed by three washes with sterile PBS and resuspension in PBS with a density of 1 × 10^8^ RBCs/mL. GC38 was diluted in PBS to concentrations ranging from 3.125 to 50 μM. For the assay, 100 µL of the RBC suspension was mixed with an equal volume of GC38 solution and incubated at 37 °C for 30 min. After incubation, the samples were centrifuged at 1500 rpm for 5 min to pellet intact RBCs. The absorbance of the supernatant was measured at 450 nm using a multi-mode microplate reader (BioTek Synergy™ H1, Winooski, VT, USA). PBS and 1% Triton X-100-treated groups served as negative and positive controls, respectively. The percentage of hemolysis was calculated by comparing the absorbance values of the samples to those of the controls. All concentrations underwent triplicate testing, with results presented as mean ± SEM.

### 4.6. Anticoagulant and Antithrombotic Efficacy of GC38 in Mouse Models and Tail Bleeding Time Assay

#### 4.6.1. Animals and Ethics

The C57BL/6J mice (male, 6–8 weeks old) were purchased from Beijing Vital River Laboratory Animal Technology Co., Ltd. (Beijing, China). All animal studies were performed under the approval of the Ethics Committee of the Laboratory Animal Welfare of the Kunming Institute of Zoology, Chinese Academy of Sciences (IACUC-RE-2025-03-010).

#### 4.6.2. FeCl_3_-Induced Carotid Artery Thrombosis Model

Male C57BL/6J mice (6–8 weeks old, n = 6 per group) were randomly assigned to groups receiving intravenous injections of PBS (vehicle control), GC38 (2 or 4 mg/kg in PBS), and heparin sodium (1000 U/kg), respectively. Ten minutes post-injection, mice were anesthetized with 2% isoflurane inhalation. The right carotid artery was surgically exposed, and endothelial injury was induced by applying a 1 × 2 mm filter paper saturated with 10% (w/v) FeCl_3_ solution for 30 s [53,54]. Real-time blood flow was monitored using a real-time laser speckle perfusion (LSP) imaging system (RWD Life Science, Shenzhen, China) with SIM BFI software (SIM BFI HR Pro, Simopto, Beijing, China). Thrombosis progression and vessel occlusion were evaluated using real-time ultrasound imaging of the region of interest at baseline (before injury), 5, and 10 min (post-injury). The perfusion unit was recorded to quantify the blood flow. Vessel occlusion was defined as a sustained reduction in perfusion units to ≤10% of baseline for at least 3 min. Data are presented as mean ± SEM.

#### 4.6.3. Stasis-Induced Deep Vein Thrombosis (DVT) Model

Male C57BL/6J mice (6–8 weeks old, n = 10 per group) were randomized to receive intravenous injections of PBS (vehicle control), GC38 (2 or 4 mg/kg), or heparin sodium (1000 U/kg) for the establishment of stasis-induced DVT models. The DVT model was induced using a mouse model of inferior vena cava (IVC) ligation [55]. Briefly, mice were anesthetized, and the IVC was exposed through a midline abdominal incision. The left proximal end of the inferior vena cava was ligated using a suture. Finally, the abdominal wall was closed carefully. At 24 h post-ligation, the IVC was resected, and thrombus formation was quantified by measuring the weight of the thrombi (n = 5). HE staining analysis of thrombus sections was performed to evaluate the extent of thrombosis (n = 5).

#### 4.6.4. Rose Bengal-Mediated Cortical Photothrombosis Stroke (CPS) Model

The Rose Bengal-mediated CPS model was employed to induce focal cerebral ischemia in mice [56]. Rose Bengal (50 mg/kg), a photosensitive dye, was administered intravenously 1 h before the surgery, followed by an intravenous injection of PBS (vehicle control), GC38 (2 or 4 mg/kg), and heparin sodium (1000 U/kg) 10 min before the surgery. Briefly, mice were anesthetized and secured in a stereotaxic frame. A midline scalp incision was made to expose the skull, followed by illumination with a 562 nm yellow-green laser (R-LG561-100-A5, RWD Life Science, Shenzhen, China) for 7 min. This process generated reactive oxygen species, resulting in endothelial damage and subsequent thrombus formation. Post-illumination, the scalp was sutured, and mice were placed on a warming pad for recovery. Cerebral ischemia severity was evaluated using the LSP imaging system, and the 15-day survival rates after ischemia induction were recorded.

#### 4.6.5. Tail Bleeding Time

The mouse tail-bleeding assay [54] was performed to evaluate the bleeding risk associated with GC38 treatment. Male C57BL/6J mice (6–8 weeks, n = 6 per group) were randomized to receive intravenous injection of PBS (negative control), GC38 (2 or 4 mg/kg in PBS), or heparin sodium (1000 U/kg, positive control). The assay was conducted 10 min after administration. Mice were anesthetized, and a 2 mm segment of the tail tip was surgically transected using a sterile scalpel. Immediately after the incision, the tail was immersed in prewarmed PBS (37 °C) to facilitate blood flow. Bleeding time was recorded from the moment of the incision until the complete cessation of bleeding (no blood flow for 60 s).

### 4.7. Statistical Analysis

Statistical analyses were performed with GraphPad Prism 10 (GraphPad Software, Inc., La Jolla, CA, USA). Data are expressed as mean ± SEM. An unpaired *t*-test was used for comparisons between two groups, while one-way analysis of variance (ANOVA) followed by Dunnett’s test was applied for multiple-group comparisons. Differences were considered significant at *p* < 0.05.

## 5. Conclusions

In conclusion, spider venom has been described as an extensive library for new and adaptive roles of secretory proteins. Through sequence similarity network analysis of de novo assembled spider venom transcriptomes and virtual screening by binding to APC, we identified GC38, the first cystine knot peptide derived from spider venom with potent anticoagulant activity. In vitro and in vivo studies demonstrate that GC38 increases APC activity by inducing conformational changes in its catalytic pocket, thereby significantly suppressing thrombus formation in both arterial and venous compartments. Crucially, GC38 has negligible cytotoxicity and nearly no hemolytic activity without prolonging bleeding time, which is a critical safety advantage over heparin sodium. For the first time, these findings have provided a new direction for the application of spider venom-derived peptides with antithrombotic activity.

## Data Availability

Data will be made available upon request.

## Supplementary Materials

The supporting information can be downloaded at: https://www.mdpi.com/article/10.3390/ijms262010154/s1.

## Abbreviations

The following abbreviations are used in this manuscript:

APC	Activated protein C
MD	Molecular dynamics
PDB	Protein Data Bank
RMSD	Root mean square deviation
RMSF	Root mean square fluctuation
MM/PBSA	Mechanics/Poisson–Boltzmann surface area
HPLC	High-performance liquid chromatography
BLI	Biolayer interferometry
ICK	Inhibitor cystine knot
PRT	Plasma recalcification time
PT	Prothrombin time
APTT	Activated partial thromboplastin time
HE staining	Hematoxylin–eosin staining
NGS	Next-generation sequencing
LSP	Laser speckle perfusion
SPPS	Solid-phase peptide synthesis
TFA	Trifluoroacetic acid
CCK-8	Cell Counting Kit 8
CPS	Cortical photothrombosis stroke
IVC	Inferior vena cava
DVT	Deep vein thrombosis
ANOVA	One-way analysis of variance
SEM	Standard error of the mean
