# Supplementary material for: Discovery of a Novel Anticoagulant Cystine Knot Peptide from Spider Venom Gland Transcriptome
Source: Int J Mol Sci. 2025 Oct 19;26(20):10154. doi: 10.3390/ijms262010154 (PMC12564639; doi:10.3390/ijms262010154)
Supplement: Supplementary file 1 [file ijms-26-10154-s001.zip › ijms-3902197-supplementary.pdf]

International Journal of *Molecular Sciences*

MDPI

# Supporting Materials

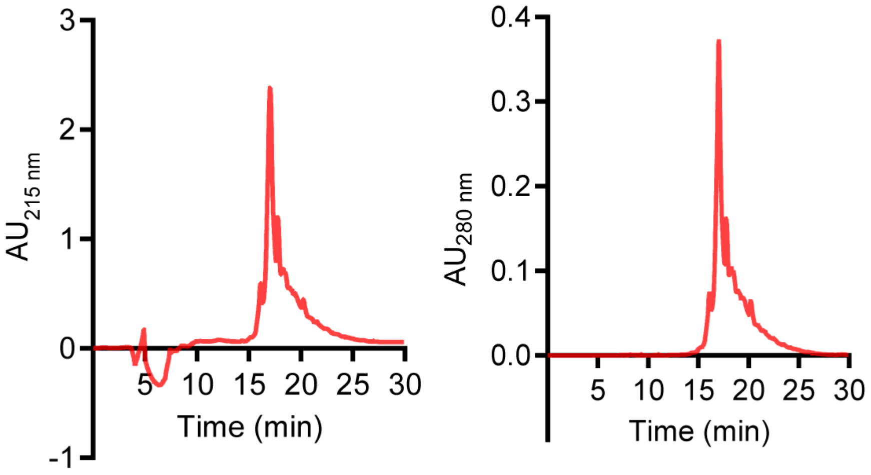


**Figure S1.** Purification of GC38 via HPLC liquid chromatogram. HPLC conditions: The gradient of acetonitrile (in 0.1% (v/v) aqueous trifluoroacetic acid) was increased from 10% to 50% over a time of 10 to 50 minutes.

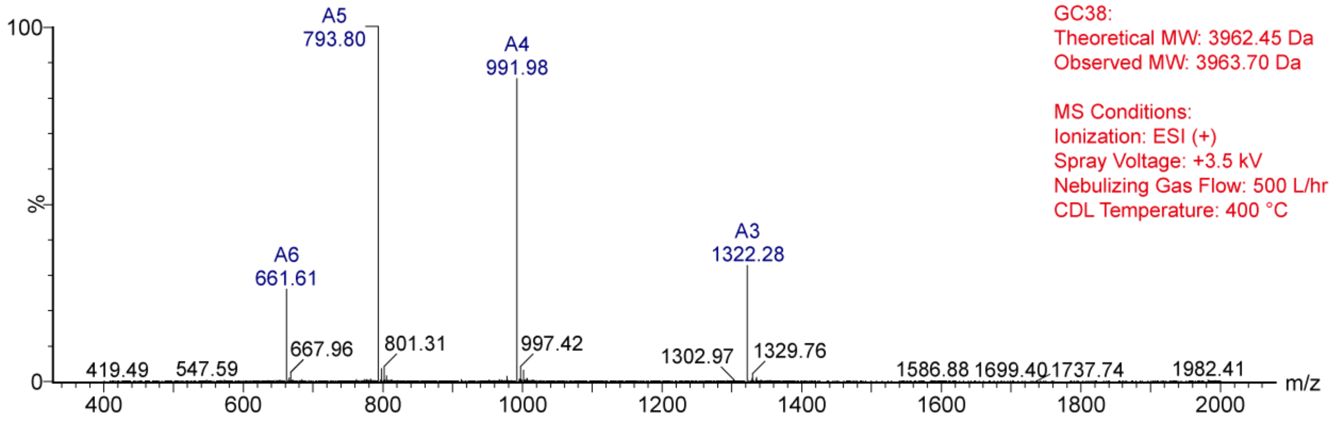


**Figure S2.** Mass spectrum analysis of GC38. Theoretical molecular weight (MW) is 3962.45 Da, while the observed MW is 3963.70 Da. The mass spectrum was obtained under the following conditions: ionization method - ESI (+), spray voltage - +3.5 kV, nebulizing gas flow - 500 L/hr, CDL temperature - 400°C. The m/z values range from 400 to 2000, with specific peaks labeled.
